# Profiling the Interaction between Human Serum Albumin and Clinically Relevant HIV Reverse Transcriptase Inhibitors

**DOI:** 10.3390/v16040491

**Published:** 2024-03-22

**Authors:** Andreia Costa-Tuna, Otávio A. Chaves, Zaida L. Almeida, Rita S. Cunha, João Pina, Carlos Serpa

**Affiliations:** 1 CQC-IMS, Department of Chemistry, University of Coimbra, Rua Larga, 3004-535 Coimbra, Portugal; andreia.dacostatuna@gmail.com (A.C.-T.); zalmeida@qui.uc.pt (Z.L.A.); anarita0016@gmail.com (R.S.C.); jpina@qui.uc.pt (J.P.); 2 Laboratory of Immunopharmacology, Centro de Pesquisa, Inovação e Vigilância em COVID-19 e Emergências Sanitárias (CPIV), Oswaldo Cruz Institute (IOC), Oswaldo Cruz Foundation (Fiocruz), Rio de Janeiro 21040-361, RJ, Brazil

**Keywords:** prodrugs, HIV reverse transcriptase inhibitors, tenofovir, HSA binding, spectroscopy techniques, in silico calculations, isothermal titration calorimetry

## Abstract

Tenofovir (TFV) is the active form of the prodrugs tenofovir disoproxil fumarate (TDF) and tenofovir alafenamide (TAF), both clinically prescribed as HIV reverse transcriptase inhibitors. The biophysical interactions between these compounds and human serum albumin (HSA), the primary carrier of exogenous compounds in the human bloodstream, have not yet been thoroughly characterized. Thus, the present study reports the interaction profile between HSA and TFV, TDF, and TAF via UV–Vis, steady-state, and time-resolved fluorescence techniques combined with isothermal titration calorimetry (ITC) and in silico calculations. A spontaneous interaction in the ground state, which does not perturb the microenvironment close to the Trp-214 residue, is classified as weak. In the case of HSA/TFV and HSA/TDF, the binding is both enthalpically and entropically driven, while for HSA/TAF, the binding is only entropically dominated. The binding constant (*K_a_*) and thermodynamic parameters obtained via ITC assays agree with those obtained using steady-state fluorescence quenching measurements, reinforcing the reliability of the data. The small internal cavity known as site I is probably the main binding pocket for TFV due to the low steric volume of the drug. In contrast, most external sites (II and III) can better accommodate TAF due to the high steric volume of this prodrug. The cross-docking approach corroborated experimental drug-displacement assays, indicating that the binding affinity of TFV and TAF might be impacted by the presence of different compounds bound to albumin. Overall, the weak binding capacity of albumin to TFV, TDF, and TAF is one of the main factors for the low residence time of these antiretrovirals in the human bloodstream; however, positive cooperativity for TAF and TDF was detected in the presence of some drugs, which might improve their residence time (pharmacokinetic profile).

## 1. Introduction

**Figure 1:**
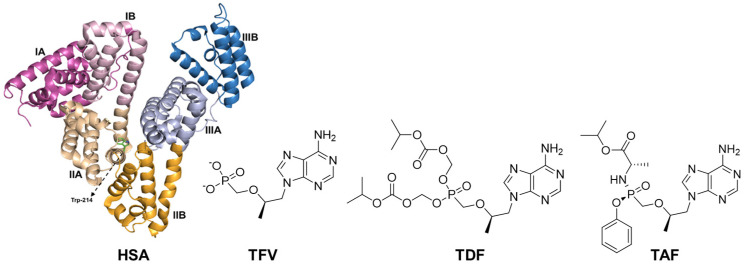
Three-dimensional structure representation of human serum albumin (HSA, PDB code: 3JRY) and chemical structures of tenofovir (TFV), tenofovir disoproxil fumarate (TDF), and tenofovir alafenamide (TAF). In the case of albumin, the different colors correspond to each subdomain, and the tryptophan (Trp) residue is highlighted in green.

The human immunodeficiency virus (HIV) is one of the most widely studied and represents one of the most prevalent and disseminated lentiviruses. As of 2022, its prevalence has escalated to encompass over 45.7 million individuals, with a noteworthy influx of 1–1.7 million new infections documented within the same year [1]. HIV belongs to the lentivirus class of retroviruses, causing chronic and often fatal diseases characterized by a slow progression of infection in the host [2]. Genomic ribonucleic acid (gRNA) serves as the genome for retroviruses and is replicated through the formation of viral DNA (vDNA) via retro-transcription, a process regulated by a viral DNA polymerase known as reverse transcriptase [3]. Antiretroviral therapy (ART) is considered one of the most effective ways to decrease the number of HIV-infected cells in the bloodstream. This therapy involves a combination of drugs, typically drawn from two or three different classes of inhibitors [4]. The present work focuses on the study of two ARTs belonging to the class of reverse transcriptase inhibitors (RTIs): tenofovir disoproxil fumarate (TDF, Figure 1) and tenofovir alafenamide (TAF, Figure 1). Both are prodrugs that, in vivo, are converted into tenofovir (TFV, Figure 1), an acyclic nucleoside phosphonate (nucleotide) analog of adenosine 5′-monophosphate [5]. The use of prodrugs aims to enhance the pharmacotherapeutic properties of the original drug, i.e., TDF permeates cells more rapidly than TFV due to its high hydrophobicity, and TAF exhibits higher biological activity than TFV or TDF, primarily due to its stability in biological matrices, including plasma’s proteins [6,7].

Human serum albumin (HSA) is the most abundant globular protein in human plasma, synthesized in the liver as a single non-glycosylated chain, with a range concentration of 35 to 50 mg/mL [8]. HSA is a monomeric multi-domain biomacromolecule with numerous functions, e.g., as a modulator of plasma oncotic pressure and carrier of a variety of endogenous and exogenous compounds until their specific targets [9,10,11,12]. The interaction between HSA and drugs is reversible, and the corresponding binding affinity is one of the main factors that determine the pharmacokinetic profile of the drugs [13]. From a structural point of view, HSA consists of 585 amino acids that form a single polypeptide chain that contains 17 pairs of disulfide bonds. The HSA structure (Figure 1) is divided into three domains, I (residues 1–194), II (residues 195–385), and III (residues 386–585), each of which features two subdomains—A with six α-helices and B with four α-helices [10,14]. HSA’s structure has three main types of binding sites: Sudlow’s site I, known as the warfarin-binding site; Sudlow’s site II, known as the benzodiazepine- or ibuprofen-binding site; and site III, known as the digitoxin-binding site, which are in subdomains IIA, IIIA, and IB, respectively [13,14,15,16].

The interaction between HSA and TFV under physiological conditions was previously reported by Shahabadi and coworkers [17]. In this case, a spontaneous interaction was detected via ground state association into subdomain IIA, stabilized mainly by hydrophobic forces. Additionally, the authors suggested one main binding site from albumin to TFV without deeply exploring the binding affinity and the effect of other compounds on the interactive profile between HSA and TFV. Since TFV is not the administered drug and there is a lack of information about the biophysical characterization of the interaction between HSA and prodrugs TDF and TAF—the biophysical characterization of HSA/TFV/TDF/TAF has not been correlated with the pharmacokinetic profile of these antiretrovirals—the present study reports the albumin–antiretroviral interaction profile via multiple spectroscopic techniques (UV–Vis, steady-state, and time-resolved fluorescence), combined with isothermal titration calorimetry (ITC) and molecular docking calculations.

## 2. Materials and Methods

### 2.1. General Materials

All reagents, including ibuprofen, digitoxin, phenylbutazone, Ludox^®^, TFV, TDF, TAF, and HSA (purity ≥ 99%, catalog number A3782), were provided by Merck KGaA company (Darmstadt, Germany) and used without further purification. The phosphate buffer solution (PBS) was prepared with 137 mM sodium chloride (NaCl), 2.7 mM potassium chloride (KCl), 8 mM disodium phosphate (Na_2_HPO_4_), and 2 mM potassium dihydrogen phosphate (KH_2_PO_4_) to achieve pH 7.4.

### 2.2. UV-Vis Measurements

The absorption spectra were recorded on an Agilent Cary 5000-UV-Vis-NIR spectrometer (Santa Clara, CA, USA) at room temperature (298 K). Three different spectra were measured in a quartz cell with a 1.0 cm optical pathlength in the 200–800 nm range with PBS as a baseline, namely non-bound HSA solution (10 μM in PBS), antiretroviral drugs (8.2, 10, and 26 μM in PBS), and HSA/antiretrovirals (with a fixed HSA concentration of 10 μM and antiretroviral concentrations of 8.2, 10, and 26 μM in PBS).

### 2.3. Steady-State Fluorescence Measurements

The steady-state fluorescence spectra were obtained in a Horiba-Jobin Yvon Fluorolog 3.2.3 (Horiba Scientific, Piscataway, NJ, USA) coupled with a thermostat cuvette holder. The measurements were obtained in the 320–350 nm range at four different temperatures (289, 296, 303, and 310 K) with an excitation wavelength (λ_exc_) of 295 nm. With a fixed concentration of 10 μM of albumin, the concentration of each drug was successively increased until final concentrations of 8.2, 9.2, 10, 13, 16, 19, 23, and 26 μM were achieved. Since TFV, TDF, and TAF do not have absorption at the excitation and maximum fluorescence emission of the biomacromolecule (295 and 340 nm, respectively), the inner filter correction was not applied. To obtain quantitative parameters describing the binding affinity between HSA and antiretrovirals, mathematical approximations from the Stern–Volmer (Equation (1)), double-logarithmic (Equation (2)), van’t Hoff (Equation (3)), and Gibbs free energy (Equation (4)) equations were used [18,19,20,21,22]:

(1) $$\frac{F_{0}}{F}=1+k_{q}\tau_{0}\left[Q\right]=1+K_{SV}$$

(2) $$\log\left(\frac{F_{0}-F}{F}\right)=logK_{b}+n\;log[Q]$$

(3) $$lnK_{SV}=-\frac{∆H{}^{\circ}}{RT}+\frac{∆S{}^{\circ}}{R}$$

(4) ∆G°=∆H°−T∆S°

where *F*_0_ and *F* are the steady-state fluorescence intensity of HSA in the absence and the presence of antiretrovirals, respectively. [*Q*] is the antiretroviral concentration, while *K_SV_* and *k_q_* are the Stern–Volmer constant and the bimolecular quenching rate constant, respectively. τ_0_ is the obtained experimental average fluorescence lifetime for HSA without antiretrovirals in PBS (around 5.06 ns in this work). *K_b_* and *n* are the binding constant and the number of binding sites, respectively. Finally, ∆*H*°, ∆*S*°, and ∆*G*° are the enthalpy, entropy, and Gibbs free energy change, respectively. *T* and *R* are the temperature (289, 296, 303, or 310 K) and gas constant (8.3145 J mol^−1^ K^−1^), respectively. Triplicate experiments were performed, and the data were analyzed with GraphPad Prism version 8.0.1 for Windows GraphPad Software (San Diego, CA, USA). The binding parameters were presented as mean ± standard deviation (SD).

### 2.4. Competitive Binding Assays

Drug-displacement assays were carried out using site probes for site I (phenylbutazone, PHEN), site II (ibuprofen, IBU), and site III (digitoxin, DIG) [21,23]. Steady-state fluorescence measurements were obtained under the same conditions described in Section 2.3 at 310 K. Each site probe was incubated with 10 μM of HSA solution (molar ratio of 1:1) before the antiretroviral additions (8.2, 9.2, 10, 13, 16, 19, 23, and 26 μM).

### 2.5. Time-Resolved Fluorescence Measurements

Time-resolved fluorescence (TRF) decays were obtained through a home-built-time-correlated single photon counting (TCSPC) apparatus previously described [24]. The decays were collected with excitation at 282 nm (HoribaJobin-Yvon-IBH nanoLED) and emission wavelength at 338 nm. The fluorescence decays for HSA (10 μM, in PBS) and HSA/TFV (drug concentrations of 8.2, 10, and 26 μM, in PBS) and HSA/TDF (prodrug concentrations of 8.2, 10, and 26 μM in PBS), as well as the instrumental response function (IRF, collected using a Ludox^®^ dispersion), were obtained using 1024 channels until 2000 counts at the maximum. Deconvolution of the fluorescence decay curves was performed using the modulation function method, as implemented by G. Striker in the SAND software version 1.0, as previously reported in the literature [25]. The average fluorescence lifetime (τ_average_) was determined following Equation (5):

(5) $$\tau_{\mathrm{average}}=\frac{\sum A_{i}\;\tau_{i}^{2}}{\sum A_{i}\;\tau_{i}}$$

where *τ_i_* is the fluorescence lifetime and *A_i_* is the pre-exponential factor.

### 2.6. Isothermal Titration Calorimetry (ITC) Measurements

The ITC experiments were performed on a Malvern MicroCal VP-Isothermal Titration Calorimetry instrument (Malvern Panalytical Ltd., Grovewood Road, Malvern, UK) at 289 K, stirring speed 459 rpm, and reference power 10 μcal/s. The titration was performed with injections of 10 μL separated by regular intervals of 450 seconds to allow equilibrium. The HSA concentration introduced in the cell was 239 μM, while the concentration of TFV or TDF introduced in the syringe was 2390 μM. All solutions were previously degassed for 10 min. The respective heat dilutions were performed and subtracted accordingly. The obtained thermograms were analyzed using Origin 7.0 software provided by Malvern. For the calculation of the binding constant (*K_a_*), number of binding sites (*n*), and thermodynamic signature of the interaction (∆*H*°, ∆*S*°, and ∆*G*°), data analysis was performed according to a model of binding to a macromolecule with one ligand binding site, by non-linear regression [26]. The calorimetric titration provides the heat changes acquired during the injection of antiretroviral into the cell containing albumin, which results in the binding isotherm curve. Thus, in this case, it is not possible to provide the error bars in the binding isotherm curve.

### 2.7. Molecular Docking Procedure

The 3D structure for HSA was obtained from Protein Data Bank (PDB) with access codes 1E7A, 2BXK, 2BXP, 2VUE, 3JRY, and 4L9K. The TFV, TDF, and TAF structure was built and energy-minimized with Spartan’14 software (Wavefunction, Inc., Irvine, CA, USA) [27] by the Density Functional Theory (DFT) method. The molecular docking calculations were performed with GOLD 2022.3.0 software (Cambridge Crystallographic Data Centre, Cambridge, CB2 1EZ, UK) [28]. The molecular docking calculations were carried out for an 8 Å radius around the selected amino acid residue present in each one of the interactive binding sites: Trp-213, Tyr-161, and Tyr-411 residues for sites I, II, and III, respectively. The score function ChemScore was used due to the lowest root mean square deviation (RMSD) value obtained by redocking studies. The 2D plots were obtained with the ProteinsPlus platform (Zentrum für Bioinformatik, Universitat Hamburg, Hamburg, Germany) [29].

## 3. Results and Discussion

### 3.1. Qualitative Binding Evaluation

UV-Vis absorption spectroscopy is a simple technique frequently applied to identify structural changes and complex formation between biomacromolecules and small compounds [30]. While biomacromolecule–ligand association in the ground state typically results in changes to the UV-Vis absorption spectrum, the absence of such alterations may indicate a lack of complex formation or suggest that the association is influenced by other phenomena, such as dynamic interactions [30,31].

**Figure 2:**
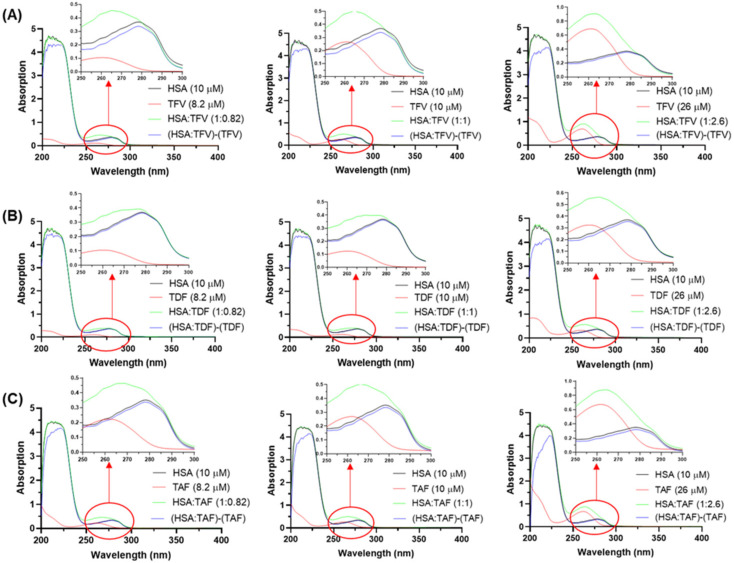
Absorption spectra of (**A**) TFV, (**B**) TDF, and (**C**) TAF in PBS (red line). The HSA spectra in PBS (black line), the interactive profile HSA/antiretrovirals (green line), and the mathematical subtraction (HSA/antiretroviral)-(antiretroviral) (blue line) are also represented. The concentrations of albumin and drugs used are indicated in the spectra.

Figure 2 depicts the UV-Vis spectra for (i) non-bound antiretrovirals, (ii) non-bound HSA, (iii) HSA/antiretroviral mixture, and (iv) the non-bound antiretroviral subtracted spectra at three different protein/drug concentration ratios (1:0.82, 1:1, and 1:2.6). The HSA spectrum has two absorption maxima: one at 220 nm, attributed to an *n*→π* transition from the carbonyl groups of the peptide bound, and the other at 280 nm, attributed to a π→π* transition associated with the aromatic amino acid residues phenylalanine (Phe), tryptophan (Trp), and tyrosine (Tyr) [18].

After the addition of the antiretrovirals to the HSA solution, an apparent hyperchromic effect was observed in the 250–300 nm range, indicating an association between albumin and the drug (green line in Figure 2). To determine if the hyperchromic effect was due to a ground state association rather than a consequence of signal addition, the contribution of the non-bound drug was subtracted from the spectrum of the complex, thus confirming a hypochromic effect that reflects a ground state association. The minor hypochromic phenomenon indicated that the respective interactions in the complexes HSA/TFV, HSA/TDF, and HSA/TAF are weak [32,33]. Additionally, the hypochromic effect (blue line in Figure 2) was also evidenced in the 200–240 nm range, indicating that TDF, TFV, and TAF, even though they bind weakly with albumin, may perturb the structural content of the protein. As expected, the increasing concentration of drugs in the HSA solution perturbed the UV signal more significantly, especially in the peptide bond region, indicating a dose-dependent binding.

### 3.2. Quantitative Binding Evaluation

**Figure 3:**
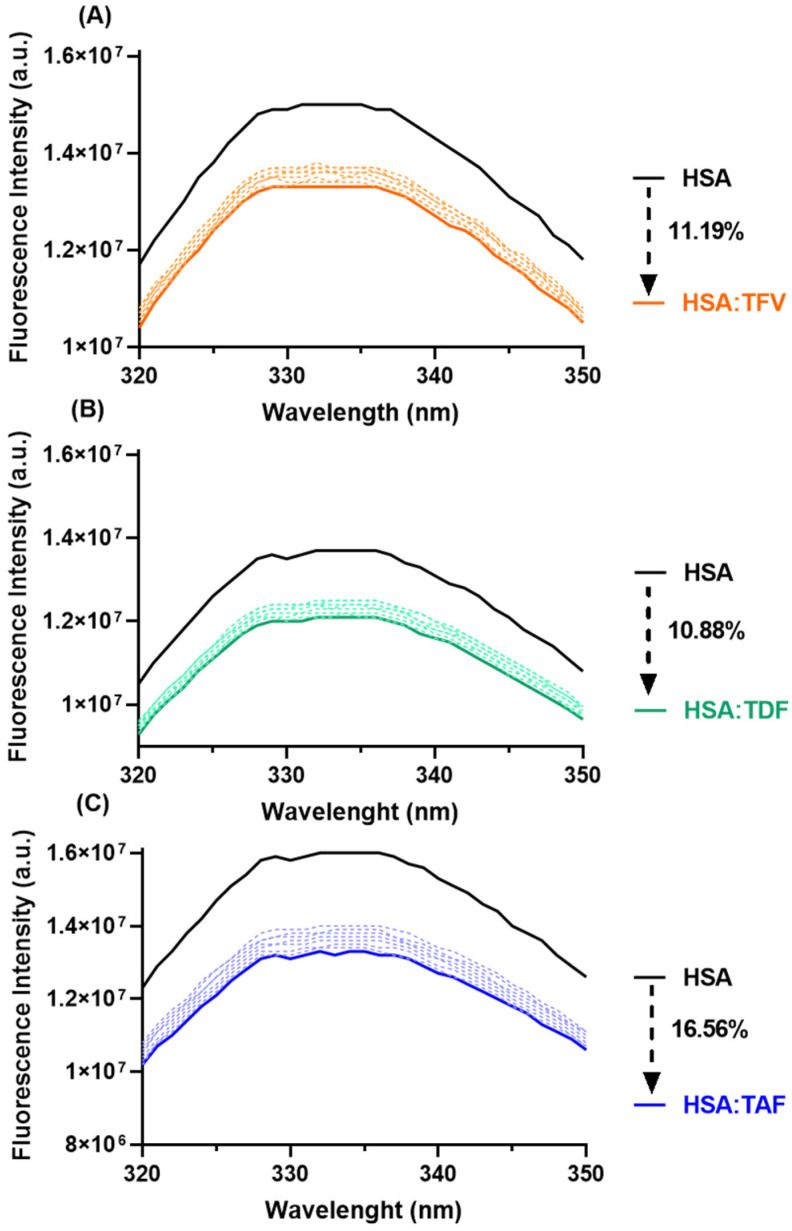
Steady-state fluorescence quenching spectra for HSA in the absence (black spectra) and in the presence of successive additions of (**A**) TFV (orange spectra), (**B**) TDF (green spectra), and (**C**) TAF (blue spectra) at 310 K in PBS. The concentration of HSA used was 10 μM, and the concentrations of antiretrovirals were 8.2, 9.2, 10, 13, 16, 19, 23, and 26 μM.

Steady-state fluorescence is the most used method for quantitatively evaluating the interaction between a fluorophore and a quencher. Thus, to better understand the binding capacity between HSA and TFV, TDF, or TAF, steady-state measurements were carried out by selectively exciting the Trp-214 residue at 295 nm [18,34]. Figure 3 depicts the steady-state fluorescence spectra for HSA in the absence and the presence of eight different concentrations of each antiretroviral at 310 K. In this case, the maximum concentration of the antiretrovirals decreased the fluorescence intensity of albumin in the range of 11–17%, with TAF being the most prominent quencher, probably due to its higher binding capacity than TFV and TDF to HSA. Additionally, no significant shift in the maximum fluorescence emission wavelength of albumin after the addition of the drugs was observed, indicating that the binding does not perturb the microenvironment close to the Trp-214 residue [18,35,36].

**Figure 4:**
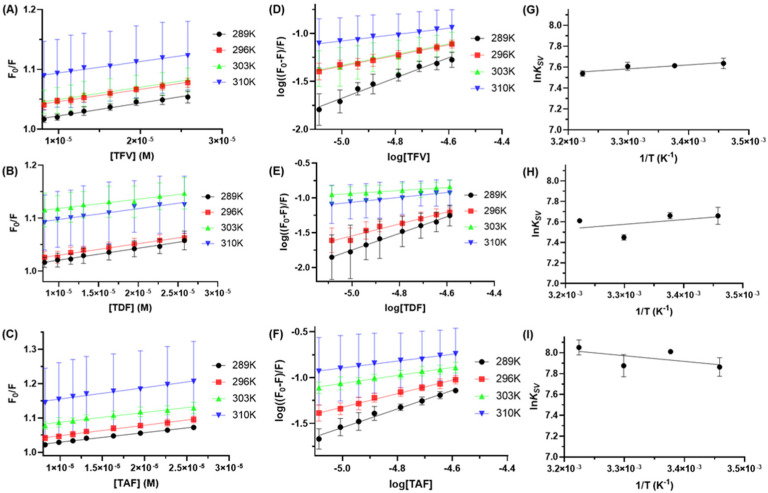
Stern–Volmer plots for the (**A**) HSA/TFV, (**B**) HSA/TDF, and (**C**) HSA/TAF interactions at 289, 296, 303, and 310 K. Double-logarithmic plots for the (**D**) HSA/TFV, (**E**) HSA/TDF, and (**F**) HSA/TAF interactions. Van’t Hoff plots based on *K_sv_* values for the (**G**) HSA/TFV, (**H**) HSA/TDF, and (**I**) HSA/TAF interactions.

The maximum intensity of the steady-state fluorescence spectra was used to determine the Stern–Volmer constant (*K_sv_*) and bimolecular quenching rate constant (*k_q_*) values. The observation of linear Stern–Volmer plots in Figure 4A–C suggests a ground state association [21], which was reinforced with *k_q_* values of about two orders of magnitude (Table 1) larger than the maximum diffusion rate constant in water (*k_diff_* ≈ 7.40 × 10^9^ M^−1^s^−1^, according to Smoluchowski–Stokes–Einstein theory at 298 K) [37]. No discernible trend in *K_sv_* values was observed with the increase in temperature. Furthermore, the large standard deviation values observed at 310 K (Figure 4) may result from heightened protein flexibility, potentially destabilizing the albumin–ligand complex. Additionally, at elevated temperatures, micro-vaporization of the PBS solution might occur.

**Figure 5:**
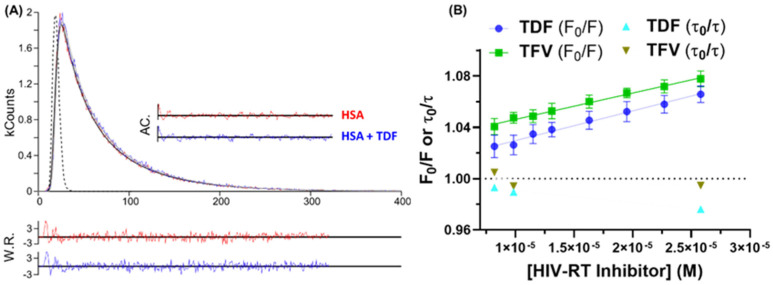
(**A**) Fluorescence decays for HSA in the absence and the presence of TDF in the molar ratio 1:2.6 at 296 K. For a better judgment of the quality of the fit, weighted residuals (WRs) and the autocorrelation function (AC) are also presented. The dashed line corresponds to the instrumental response function (IRF). (**B**) Stern–Volmer plots based on steady-state and time-resolved fluorescence data for HSA/TFV and HSA/TDF at 296 K.

To clarify the origin of the main fluorescence quenching mechanism operating in the HSA/antiretroviral interaction, time-resolved fluorescence measurements were carried out in the absence and presence of the drug TFV or prodrug TDF in the stoichiometric albumin-to-antiretroviral ratios of 1:0.82, 1:1, and 1:2.6. Since the prodrugs TDF and TAF showed similar quenching parameters (Table 1), time-resolved fluorescence studies were conducted for just one of the two prodrugs. Similar fluorescence decay lifetimes were obtained for HSA, HSA/TFV, and HSA/TDF using different proportions of antiretrovirals, as shown in Table 2 and Figure 5A, which depicts the time-resolved fluorescence decays for HSA and HSA/TDF at the highest ligand concentration (1:2.6). As summarized in Table 2, albumin presents two characteristic fluorescence lifetimes with an average lifetime of about 5.06 ns, in full agreement with literature data [38,39,40]. Since the fluorescence lifetimes for HSA did not change significantly in the presence of TFV (drug) and TDF (prodrug) and both Stern−Volmer plots for fluorescence intensities (F_0_/F) and lifetimes (τ_0_/τ) are linear with τ_0_/τ approximately equal to unity (Figure 5B), it can be stated that the quenching process occurs through a static mechanism [18], confirming the results obtained in the steady-state fluorescence measurements.

For a purely ground state association, selectively exciting one fluorophore indicates one binding site of albumin to one ligand [18,19], a notion reinforced by the binding site number (*n*) values close to unity (Figure 4D–F and Table 1) [40]. Additionally, in a static quenching mechanism, the *K_SV_* values also provide information about the binding affinity [18,19]. Given that the *K_SV_* values are around 10^3^ M^−1^, a weak interaction between serum albumin and TFV, TDF, and TAF is indicated [13]. This is likely to be one of the main reasons for the short residence time of these compounds in the human bloodstream [5]. The results agree with the slight changes in the UV-Vis spectra described in Section 3.1. Additionally, despite Shahabadi and coworkers [17] not having discussed the binding affinity between HSA and TFV, the reported quantitative values agree with those obtained here.

The concentration of albumin in human plasma typically ranges from 35 to 50 mg/mL [8], while the maximum plasma concentration of TFV, TDF, and TAF is about 27.9, 300, and 104 ng/mL, respectively [41,42]. In other words, in in vivo conditions, the albumin concentration is around 100,000-fold higher than the concentration of the antiretrovirals. Since the binding capacity of the antiretrovirals to albumin is weak and the fluorescence quenching of albumin depends on the capacity of TFV, TDF, and TAF to interact with the fluorophore (in a static quenching mechanism), we have used concentrations of antiretrovirals below, equivalent to, and higher than the concentration of albumin used to achieve feasible fluorescence quenching and improve sensibility.

The intermolecular interaction between the ligand and HSA may involve several different forces, such as van der Waals, hydrogen bonding, and hydrophobic networks. These forces are related to changes in the enthalpy and entropy (Δ*H*° and Δ*S*°, respectively) values. Table 1 summarizes the thermodynamic parameters for HSA/TFV, HSA/TDF, and HSA/TAF using the van’t Hoff approach (Figure 4G–I). According to the report of Ross and Subramanian [43], the negative and positive Δ*H*° and Δ*S*° values, respectively, for HSA/TFV and HSA/TDF indicate ionic interactions as one of the critical forces stabilizing the complex formation. On the other hand, for HSA/TAF, both positive values for Δ*H*° and Δ*S*° are indicative of substantial contributions from both ionic and hydrophobic forces. Hydrophobic interactions were probably detected for TAF due to the decrease in the number of oxygen atoms in the chemical structure of this compound as well as the incorporation of a benzyl moiety. Overall, for all antiretrovirals under study, the Gibbs free energy change (ΔG°) values are negative, agreeing with the spontaneity of the binding. Comparing the obtained thermodynamic results with those determined by Shahabadi and coworkers [17] for HSA/TFV, the reported Δ*H*° value (149.85 kJmol^−1^) is not in the same trend obtained in the current study, while the reported Δ*S*° value (0.576 kJmol^−1^K^−1^) is about 10-fold larger than that obtained here. The discrepancy may be a consequence of not having determined the main fluorescence quenching mechanism, thus leading Shahabadi and coworkers to consider a double-logarithmic approach instead of the Stern–Volmer approximation for determining the binding affinity which was subsequently used for the determination of the thermodynamic parameters.

From previous reports on the design of TFV, TDF, and TAF [6,7], it is known that the clinical use of prodrugs (TDF and TAF) aims to enhance the pharmacotherapeutic properties of the original drug (TFV), e.g., improvement of the cell permeation and stability in biological matrices. In this sense, the differences in both thermodynamic and binding affinity of TAF compared with TFV and TDF indicate that the interactive profile HSA/TAF corroborates with literature reports that TAF exhibits greater biological activity in comparison to TFV or TDF, primarily due to its stability in biological matrices, including plasma proteins [6,7].

### 3.3. Isothermal Titration Calorimetry (ITC) Analysis

ITC is a biophysical technique based on the measurement of heat absorbed or released during a host–guest interaction, providing an accurate, rapid, and label-free measurement of the thermodynamics of molecular interactions [44,45]. Thus, to better characterize the binding affinity between HSA and antiretrovirals compared with the obtained experimental spectroscopic data described above, ITC experiments were carried out for the drug TFV and prodrug TDF at 289 K. Since the prodrugs TDF and TAF showed similar quenching parameters (Table 1), ITC studies were conducted for just one of the two prodrugs. Additionally, steady-state fluorescence measurements detected weak binding capacity for albumin–antiretrovirals; thus, in the ITC assays, the maximum antiretroviral concentration soluble in PBS medium (10-fold higher than albumin concentration) was required, and a temperature of 289 K was used to inhibit the probability of albumin aggregation [46,47,48].

**Figure 6:**
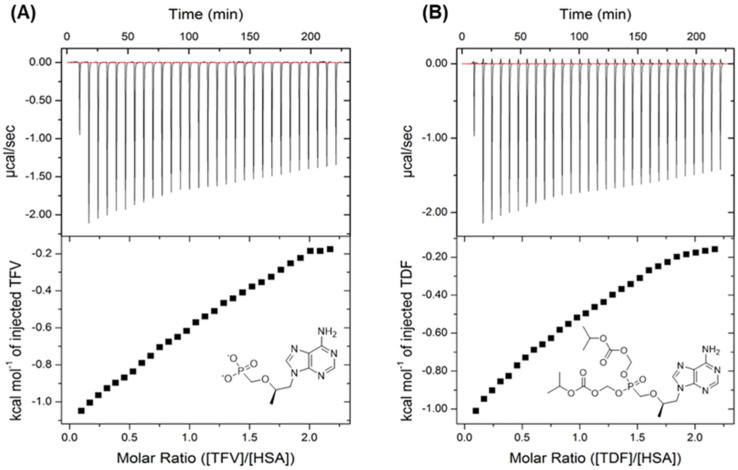
Calorimetric titration of (**A**) HSA/TFV and (**B**) HSA/TDF at 289 K. The top panels display the heat changes acquired during the injection of antiretroviral into a cell containing HSA. The bottom panels show the binding isotherm curve that corresponds to the data in the top panels.

Figure 6 depicts the ITC plots for the HSA/TFV and HSA/TDF interactions. Negative peaks were observed in both plots, indicating an exothermic interaction, in agreement with the Δ*H*° value from the steady-state fluorescence studies (heat release). As can be seen in ITC thermograms, the heat release observed is very small, and the titration resulted in a weak curve due to the low binding affinity of TFV and TDF with albumin, as expected from the observed spectroscopic trend.

The binding constant (*K_a_*), the number of binding sites (*n*), and thermodynamic parameters (∆*H*°, ∆*S*°, and ∆*G*°) were determined from ITC thermograms, and the results are summarized in Table 3. The *K_a_* values are comparable with the *K_SV_* values, indicating a weak binding capacity between albumin and the antiretrovirals under study. Additionally, the *n* values are close to unity, corroborating with the proposed 1:1 stoichiometry as previously mentioned. Interestingly, the ∆*H*° and ∆*S*° values have the same signs and similar quantitative values compared with those obtained from the steady-state fluorescence data (Section 3.2), confirming that the HSA/TFV and HSA/TDF interactions are both enthalpically and entropically driven, consistent with spontaneous albumin/antiretroviral binding (∆*G*° < 0).

### 3.4. Identification of the Main Binding Site—Experimental and In Silico

**Figure 7:**
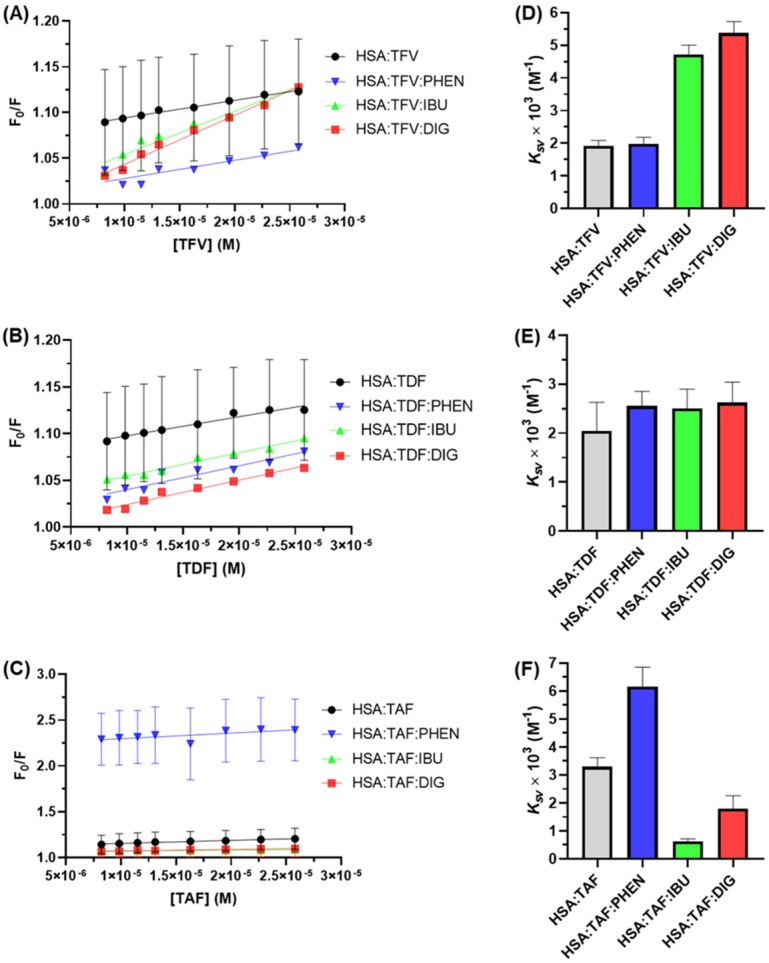
Stern–Volmer plots for the interaction between HSA and (**A**) TFV, (**B**) TDF, and (**C**) TAF at 310 K in the absence and presence of the site markers phenylbutazone (PHEN), ibuprofen (IBU), and digitoxin (DIG). The *K_SV_* values were obtained from the Stern–Volmer plots for (**D**) HSA/TFV, (**E**) HSA/TDF, and (**F**) HSA/TAF at 310 K.

According to Sudlow and co-workers [49], HSA has two prominent binding cavities located in hydrophobic regions: site I (the phenylbutazone binding site located in the subdomain IIA) and site II (the ibuprofen binding site located in the subdomain IIIA) (Figure 1). Additionally, a third binding site, namely site III (digitoxin binding site in the subdomain IB) was later reported [36,50,51] (Figure 1). To determine the main binding site of albumin to TFV, TDF, and TAF, drug-displacement assays were carried out in the presence of phenylbutazone (PHEN), ibuprofen (IBU), or digitoxin (DIG) at 310 K. Stern–Volmer plots and the corresponding *K_SV_* values are depicted in Figure 7.

In the case of HSA/TFV, there is a positive cooperativity in the presence of the commercial drugs IBU and DIG, while for HSA/TAF, the positive cooperativity occurs in the presence of PHEN, and for HSA/TDF, the *K_SV_* values did not change significantly in the presence of PHEN, IBU, or DIG. In positive cooperativity, the binding of the first ligand molecule might increase the apparent affinity of the receptor and consequently increase the likelihood of the second ligand molecule binding, probably because of induced conformational changes in the structure of the receptor [52]. In this case, besides the results indicating the main binding site of the antiretrovirals, they highlight the improvement in the residence time of TAF and TDF in the bloodstream with the coadministration of other types of drugs.

Although TFV, TDF, and TAF exhibit slight differences in binding affinity to albumin, the distinctions in their chemical structures—specifically, variations in the structural units connected to the phosphate group—result in differences in the main binding sites. For instance, TAF might interact with subdomains IIIA or IB, while TDF binds indiscriminately to one of the three studied binding sites, and TFV might interact with subdomain IIA. The small internal cavity known as site I is probably the main binding pocket for TFV due to the low steric volume of this drug. In contrast, most external sites (II and III) can better accommodate TAF due to the larger steric volume of this prodrug.

**Figure 8:**
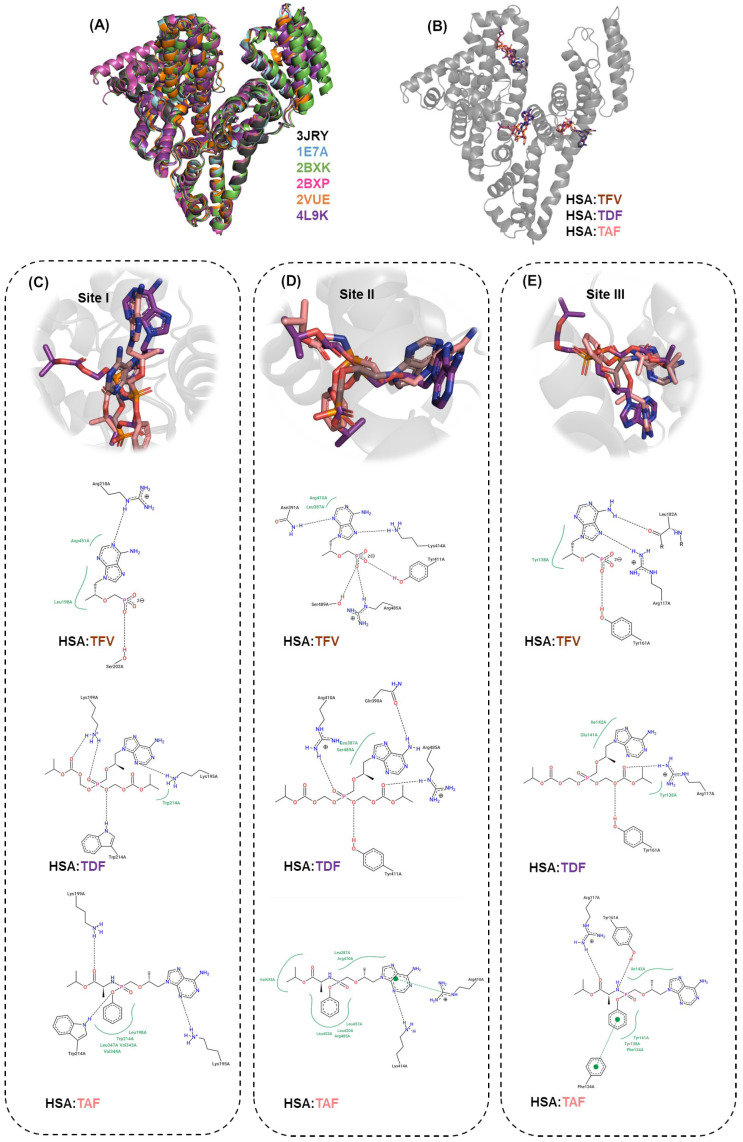
(**A**) Superposition of the three-dimensional crystallographic structure of HSA. (**B**) Superposition of the best docking pose of HSA/TFV, HSA/TDF, and HSA/TAF with the corresponding zoom representation and interactive profile of (**C**) site I, (**D**) site II, and (**E**) site III. The amino acid residues that interact hydrophobically with the antiretrovirals are in green, while interactions via hydrogen bonds are shown by black dots. Hydrogen atoms were omitted for better interpretation.

To offer an atomic point of view of the interactive profile between albumin and the antiretrovirals under study, in silico calculations via molecular docking were carried out at pH 7.4. Unlike most of the literature reported on molecular docking calculations for the binding of albumins to small organic compounds, the present study has primarily explored the cross-docking approach [53] to suggest the impact of small endogenous and exogenous compounds on the binding capacity of TFV, TDF, and TAF, thus being more realistic within a biological context. In this sense, the superposition of the three-dimensional structures of HSA without (PDB code: 3JRY) and with certain metabolites and commercial drugs (PDB codes: 1E7A, 2BXK, 2BXP, 2VUE, and 4L9K), such as camptothecin (anticancer drug), indomethacin (anti-inflammatory drug), azapropazone (anti-inflammatory drug), myristic acid (one of the most abundant fatty acids in milk fat), propofol (anesthetic drug), phenylbutazone (anti-inflammatory drug), and 4*Z*,15*E*-bilirubin-IX-alpha (compound that occurs in the normal catabolic pathway that breaks down heme in vertebrates), yielded a root mean square deviation (RMSD) value in the range of 0.638–2.641 Å, suggesting a dependence of the three-dimensional conformation of albumin on the nature of the ligand that was crystallized with the protein (Figure 8A).

The docking score values (dimensionless) are summarized in Table 4. For albumin without any co-crystallized compound (PDB code: 3JRY), the highest docking score value was obtained from site I for TFV and site III for both TDF and TAF. These results align with the experimental competitive binding assays, which indicated that the high steric volume of the prodrugs under study primarily drives the binding to an external binding pocket. Interestingly, in silico calculations also revealed that the conformation of albumin induced by the crystallized ligands might impact the main interactive pocket of albumin, corroborating with the competitive binding assays (cooperative phenomenon). For example, the presence of propofol (which selectively binds into subdomain IIIA, PDB code: 1E7A) decreased the binding affinity of TAF to subdomains IIIA and IB, while increasing the binding affinity to subdomain IIA. On the other hand, the propofol increased the binding affinity of TFV to sites II and III (positive cooperativity), while not significantly changing the binding affinity of TDF.

The molecular docking results also suggest that the adenine moiety of the antiretrovirals under study binds similarly to subdomains IIA and IIIA, assuming different binding poses mainly within subdomain IB (Figure 8B–E). This also explains the differences in the binding capacity between the drug TFV and the prodrugs TDF and TAF. Finally, hydrogen bonds and hydrophobic forces (Figure 8C–E) were detected as the main intermolecular interactions responsible for the association of albumin with antiretrovirals. For example, in the case of HSA/TDF at site II, the oxygens of the negatively charged phosphate group of the antiretroviral structure are potential acceptors for hydrogen bonds with the positively charged Arg-485 residue, while at sites I and III, the hydroxyl groups from Ser-202 and Tyr-161 residues, respectively, are potential donors for hydrogen bonds. For all antiretrovirals, regardless of the binding site, the positively charged amino acid residues are important for the stability of the association with albumin due to the hydrogen bond interactions. Interestingly, among the antiretrovirals under study, TAF showed a greater number of connecting points and possibilities for hydrophobic interactions, consistent with its higher experimental binding affinity compared to TFV and TDF, and with its reported stability in biological matrices, including plasma proteins [6,7].

Overall, the obtained experimental and in silico results offer a biophysical perspective on the interaction of albumin with antiretrovirals, allowing a better understanding of the low residence time of TFV, TDF, and TAF in the human bloodstream. However, albumin is not the only endogenous carrier of drugs; lipoproteins may also interact with different compounds [54,55]. Therefore, it will be interesting to evaluate the interaction between lipoproteins and antiretrovirals in future studies. Additionally, the percentage of non-bound albumin in the human bloodstream is not very high compared to the percentage of albumin bound with different endogenous compounds, such as fatty acids [56]. Thus, future work on the influence of fatty acids on the association of albumin with antiretrovirals will also be an interesting approach to better correlate blood fat levels with the pharmacokinetics of antiretrovirals.

## 4. Conclusions

The interaction between HSA and three HIV reverse transcriptase inhibitors, one drug (TFV) and two prodrugs (TDF and TAF), is entropically driven (Δ*S*° > 0) and occurs spontaneously (Δ*G*° < 0). However, in the case of HSA/TFV and HSA/TDF, there is a significant contribution from Δ*H*° (enthalpically driven), indicating ionic interactions as one of the critical forces for stabilizing the formation of the complex. The main fluorescence quenching mechanism of albumin induced by the antiretroviral compounds is static, indicating a ground state association. Thus, the *K_SV_* value not only expresses the photophysical behavior but also estimates the binding affinity, which is weak (10^3^ M^−1^). The binding constant (*K_a_*) and thermodynamic parameters obtained via ITC assays agree with those obtained with steady-state fluorescence quenching measurements, reinforcing the reliability of the data. There is an interaction in the molar ratio of 1:1, i.e., one molecule of antiretroviral interacts with just one molecule of albumin, and following the drug-displacement assays, for HSA/TFV, there is positive cooperativity in the presence of the commercial drugs ibuprofen and digitoxin, while for HSA/TAF, positive cooperativity occurs only in the presence of phenylbutazone. For HSA/TDF, no cooperativity has been detected in the presence of phenylbutazone, ibuprofen, or digitoxin. The cross-docking approach corroborated experimental drug-displacement assays, indicating that the binding affinity of TFV and TAF might be impacted by the presence of different compounds bound to albumin. Overall, the weak binding capacity of TFV, TDF, and TAF is one of the main factors for the low residence time of these compounds in the human bloodstream. However, the positive cooperativity observed for TAF and TDF in the presence of certain drugs potentially improves their residence time and pharmacokinetic profile.

## Figures and Tables

**Table 1:** Steady-state fluorescence quenching parameters for the HSA/antiretroviral interaction at four different temperatures in PBS.

System	T (K)	K_sv_ (×10^3^) (M^−1^)	k_q_ (×10^11^) ^1^ (M^−1^s^−1^)	n	${∆H}^{0}$ (kJmol^−1^)	${∆S}^{0}$ (kJmol^−1^K^−1^)	${∆G}^{0}$ (kJmol^−1^)
HSA/TFV	289	2.15 ± 0.20	4.25 ± 0.40	1.06 ± 0.07	−3.53 ± 1.21	0.052 ± 0.004	−18.4
	296	2.05 ± 0.20	4.05 ± 0.40	0.56 ± 0.02			−18.8
	303	2.07 ± 0.62	4.09 ± 0.59	0.56 ± 0.03			−19.2
	310	1.92 ± 0.17	3.79 ± 0.34	0.33 ± 0.01			−19.5
HSA/TDF	289	2.25 ± 0.41	4.45 ± 0.47	1.19 ± 0.26	−5.44 ± 1.18	0.045 ± 0.017	−18.5
	296	2.17 ± 0.30	4.29 ± 0.43	0.86 ± 0.13			−18.8
	303	1.75 ± 0.83	3.46 ± 0.66	0.21 ± 0.11			−19.1
	310	2.04 ± 0.59	4.03 ± 0.56	0.35 ± 0.24			−19.5
HSA/TAF	289	2.77 ± 0.18	5.47 ± 0.36	1.01 ± 0.03	4.82 ± 1.10	0.083 ± 0.010	−19.1
	296	3.04 ± 0.25	6.01 ± 0.41	0.74 ± 0.02			−19.6
	303	2.84 ± 0.38	5.61 ± 0.45	0.44 ± 0.02			−20.2
	310	3.30 ± 0.32	6.52 ± 0.44	0.37 ± 0.02			−20.8

^1^ Using the τ_average_ obtained in this work for non-bound HSA (5.06 ns).

**Table 2:** Fluorescence lifetimes (τ_1_ and τ_2_), average lifetime (τ_average_), pre-exponential factors (A_1_ and A_2_), and relative contribution (%Rel) for HSA, HSA/TFV, and HSA/TDF.

System	Molar Ratio	τ_1_ (ns)	τ_2_ (ns)	A_1_	A_2_	%Rel (τ_1_)	%Rel (τ_2_)	τ_average_ (ns)
HSA/TFV	1:0	2.18	6.00	0.488	0.512	26	74	5.01
	1:0.82	2.41	6.17	0.521	0.479	30	70	5.04
	1:1	2.24	6.05	0.485	0.515	26	74	5.06
	1:2.6	2.15	6.07	0.477	0.523	24	76	5.13
HSA/TDF	1:0	2.26	6.16	0.504	0.496	27	73	5.11
	1:0.82	2.26	6.18	0.513	0.487	28	72	5.08
	1:1	2.29	6.30	0.534	0.466	29	71	5.14
	1:2.6	2.60	6.50	0.573	0.427	35	65	5.14

**Table 3:** The binding constant (*K_a_*), the number of binding sites (*n*), and thermodynamic values for HSA/TFV and HSA/TDF obtained by ITC at 289 K.

System	K_a_ (×10^3^) (M^−1^)	n	∆H° (kJmol^−1^)	∆S° (kJmol^−1^K^−1^)	∆G° (kJmol^−1^)
HSA/TFV	12.3 ± 1.0	1.24 ± 0.02	−5.54 ± 0.15	0.059	−22.6
HSA/TDF	9.60 ± 0.84	1.05 ± 0.24	−5.94 ± 0.24	0.056	−22.1

**Table 4:** Docking score values (dimensionless) for the HSA/TFV, HSA/TDF, and HSA/TAF interactions.

	HSA/TFV			HSA/TDF			HSA/TAF		
Template (PDB Code)	Site I	Site II	Site III	Site I	Site II	Site III	Site I	Site II	Site III
3JRY	17.7	15.1	16.1	27.8	20.9	28.1	26.2	28.3	29.5
1E7A	17.0	18.0	19.4	26.4	21.8	27.1	28.9	25.0	26.1
2BXK	20.0	17.6	20.8	33.3	31.2	31.4	35.9	31.7	33.8
2BXP	17.7	17.2	20.7	30.8	28.7	31.3	29.5	31.2	33.7
2VUE	18.1	19.7	19.5	28.8	27.3	31.3	26.5	27.1	34.2
4L9K	19.1	15.8	20.5	26.4	24.0	28.8	29.0	24.7	34.0

## Data Availability

All data are contained within the article.
